# Mortality in Schizophrenia and Other Psychoses: A 10-Year Follow-up of the ӔSOP First-Episode Cohort

**DOI:** 10.1093/schbul/sbu138

**Published:** 2014-09-27

**Authors:** Ulrich Reininghaus, Rina Dutta, Paola Dazzan, Gillian A. Doody, Paul Fearon, Julia Lappin, Margaret Heslin, Adanna Onyejiaka, Kim Donoghue, Ben Lomas, James B. Kirkbride, Robin M. Murray, Tim Croudace, Craig Morgan, Peter B. Jones

**Affiliations:** ^1^Centre for Epidemiology and Public Health, Health Service and Population Research Department, Institute of Psychiatry, King’s College, London, UK;; ^2^Department of Psychiatry and Psychology, School for Mental Health and Neuroscience, Maastricht University, Maastricht, The Netherlands;; ^3^Department of Psychiatry, University of Cambridge, National Institute for Health Research (NIHR) Cambridge Biomedical Research Centre and NIHR Collaboration for Leadership in Applied Health Research & Care, Cambridge, UK;; ^4^Department of Psychological Medicine, Institute of Psychiatry, King’s College, London, UK;; ^5^NIHR Mental Health Biomedical Research Centre at South London and Maudsley NHS Foundation Trust and King’s College, London, UK;; ^6^Psychosis Studies Department, Institute of Psychiatry, King’s College, London, UK;; ^7^Division of Psychiatry and Applied Psychology, University of Nottingham, Nottingham, UK;; ^8^Department of Psychiatry, Trinity College, Dublin, Ireland;; ^9^Department of Psychiatry, University of New South Wales, Sydney, NSW, Australia;; ^10^Centre for Economics of Mental and Physical Health, Health Service and Population Research Department, Institute of Psychiatry, King’s College, London, UK;; ^11^Addictions Department, Institute of Psychiatry, King’s College, London, UK;; ^12^Division of Psychiatry, University College London, London, UK;; ^13^Department of Health Sciences, University of York, York, UK

**Keywords:** schizophrenia/, mortality/, psychosis/, risk factors

## Abstract

The excess mortality in people with psychotic disorders is a major public health concern, but little is known about the clinical and social risk factors which may predict this health inequality and help inform preventative strategies. We aimed to investigate mortality in a large epidemiologically characterized cohort of individuals with first-episode psychosis compared with the general population and to determine clinical and social risk factors for premature death. All 557 individuals with first-episode psychosis initially identified in 2 areas (Southeast London and Nottinghamshire, United Kingdom) were traced over a 10-year period in the ӔSOP-10 study. Compared with the general population, all-cause (standardized mortality ratio [SMR] 3.6, 95% confidence interval [CI] 2.6–4.9), natural-cause (SMR 1.7, 95% CI 1.0–2.7) and unnatural-cause (SMR 13.3, 95% CI 8.7–20.4) mortality was very high. Illicit drug use was associated with an increased risk of all-cause mortality (adj. rate ratio [RR] 2.31, 95% CI 1.06–5.03). Risk of natural-cause mortality increased with a longer time to first remission (adj. RR 6.61, 95% CI 1.33–32.77). Family involvement at first contact strongly reduced risk of unnatural-cause mortality (adj. RR 0.09, 95% CI 0.01–0.69). Our findings suggest that the mortality gap in people with psychotic disorders remains huge and may be wider for unnatural-cause mortality than previously reported. Efforts should now focus on further understanding and targeting these tractable clinical and social risk factors of excess mortality. Early intervention and dual diagnosis services may play a key role in achieving more rapid remission and carer involvement and addressing substance use problems to reduce excess mortality in psychosis.

## Introduction

People with schizophrenia and other psychotic disorders die earlier than their peers in the general population, with recent estimates suggesting by around 15–20 years.^1–10^ Although life expectancy in the general population has risen over recent decades in developed countries,^11^ it is unclear whether the mortality gap for people with psychotic disorder has widened or narrowed.^4,5,9,12–16^ Evidence as to whether excess mortality is predominantly due to unnatural or natural causes of death remains equivocal.^2,7,9,12^ In addition, mortality has rarely been investigated in unselected cohorts of first-episode cases of all psychotic disorders. Further, most studies conducted to date have compared mortality in psychosis with that in the national rather than local population,^9^ thereby ignoring geographical variation in mortality rates.^17,18^ A number of factors have been proposed to account for the mortality gap such as smoking, alcohol use, obesity, and other unhealthy lifestyle factors that increase risk of a broad range of physical conditions, limited access to healthcare, poorer quality of healthcare received, or adverse side effects of second-generation antipsychotic medication.^9,16,19–24^ Conflicting results have been recently reported as to whether illicit drug use contributes to excess mortality in psychosis.^2,25,26^ Overall, evidence on modifiable clinical and social risk factors which may predict premature death in this population remains very limited.

We aimed to: (1) investigate mortality in a cohort of 557 individuals with first-episode psychosis, who initially presented to mental health services within defined catchment areas of the Aetiology and Ethnicity in Schizophrenia and Other Psychoses (ӔSOP) study and subsequently traced over an approximately 10-year period in the ӔSOP-10 study; (2) compare mortality in this cohort with that in the local general population; and (3) investigate baseline clinical and social factors associated with an increased risk of premature death.

## Methods

### Sample

This study forms part of ӔSOP-10, a 10-year follow-up study of a cohort of 557 individuals with a first episode of psychosis initially identified in the 2 centers (ie, Southeast London, Nottinghamshire, United Kingdom) of the ӔSOP study.^27^ All patients with a first episode of psychosis who presented to mental health services within defined catchment areas in Southeast London and Nottinghamshire were screened for inclusion at baseline. This yielded a sample of *n* = 532 incident cases. We included 25 additional cases in ӔSOP-10 identified as part of the MRI data collection at baseline. The study received full ethical approval from the relevant local research ethics committees. Full details of the methods of ӔSOP-10 have been reported by Morgan *et al*.^28^

### Case-Tracing Procedure

We identified all occurrences of death and emigration in the cohort over a combined total of 5183.9 years of follow-up until December 12, 2012 (mean length of follow-up 10.0 years, *SD* = 2.3) via a person-tracing procedure conducted on our behalf by the Office for National Statistics (ONS) for England and Wales and the General Register Office (GRO) for Scotland using name, sex, date of birth, and last known address. For all identified deaths, principal underlying causes of death were determined according to the International Classification of Diseases, 10th revision (ICD-10),^29^ as recorded on copies of death certificates obtained from ONS. We grouped these into 3 broad categories (using ICD-10 codes): natural causes to refer to the disease which initiated the train of events directly leading to death^29^ (A00–Q99), unnatural (or external) causes to refer to the circumstances of the accident or violence which produced the fatal injury^29^ (U50.9, V01–Y89), and unknown causes to refer to symptoms, signs, and abnormal clinical and laboratory findings, not elsewhere classified^29^ (R00–R99). Unnatural causes of death included accidents (V01–X59) and suicide (X60–X84 and Y10–Y34). Consistent with the classification of causes of death by ONS, both intentional self-harm (X60–X84) and events of undetermined intent (Y10–Y34) were coded as suicide. The underlying cause of death recorded on copies of death certificates was further ascertained based on extensive information collated from clinical records at follow-up using an extended version of the World Health Organization (WHO) Life Chart.^28,30^

### Data Collection

Detailed information on sociodemographic characteristics (including sex, age, and ethnicity), clinical presentation (including diagnosis, duration of untreated psychosis (DUP), and illicit drug use in past year), and social factors (including education, employment, involvement of family at first contact with mental health services) was collected at baseline.^31^ In the extended version of the WHO Life Chart,^28,30^ data on time to first remission were collected at follow-up. Remission was defined as an absence of clinical psychotic symptoms for a period of at least 6 months.^28,32^ Data on all-, natural-, and unnatural-cause mortality rates (in the population at risk) and population estimates, stratified by sex, age band, year, and the Census Area Statistics (CAS) wards in Lambeth and Southwark in Southeast London (33 CAS wards) and Nottinghamshire (95 CAS wards), in which cases were initially identified, were obtained from ONS for the duration of the follow-up period.

### Data Analysis

We calculated crude mortality rates for all causes, natural causes, unnatural causes, and unknown causes of death per 100 000 person-years by baseline sociodemographic characteristics for people with first-episode psychosis. We constructed Kaplan–Meier plots and used Cox regression to inspect variation in risk of death over time according to sociodemographic, clinical, and social characteristics. Date of first presentation to services was used as the entry point and date of death, last contact, date of emigration, or the end of follow-up as the end point, whichever came sooner. Log-rank tests were used to examine whether probability of death over time varied by sociodemographic, clinical, and social characteristics. Poisson regression modeling was conducted to quantify the effect of these characteristics on risk of all-, natural-, and unnatural-cause mortality in people with first-episode psychosis while controlling for potential confounders based on (a) the level of change in the magnitude of effect of interest and (b) whether potential confounders were associated with the outcome (by examining whether adding variables to the model improved model fit [*P* <.05] using likelihood ratio tests [LRTs] to minimize problems of overfitted models with unstable parameter estimates). Finally, we employed indirect standardization to compare mortality risk in people with first-episode psychosis with the risk in the local general population. Standardized mortality ratios (SMRs) and 95% confidence intervals (CIs) for all, natural, and unnatural causes of death were calculated based on the observed number of deaths in the cohort and the expected number of deaths for each study center by age band and sex derived from ONS all-, natural-, and unnatural-cause mortality rates (in the population at risk) and population estimates. All analyses were conducted using Stata version 12.^33^

## Results

### Mortality in the Cohort

Of the 557 cases with first-episode psychosis identified at baseline, 8 were excluded based on additional diagnostic information not available at baseline. Of the remaining 549 cases, 39 (7.1% of the sample, 717.3 per 100 000 person-years) cases had died, 15 (2.7%, 275.9 per 100 000 person-years) due to natural causes, 21 (3.8%, 386.3 per 100 000 person-years) due to unnatural causes, and 3 (0.6%, 55.2 per 100 000 person-years) due to unknown causes of death. Cases with natural causes of death predominantly died from diseases of the digestive system (*n* = 7, 1.3%, 128.8 per 100 000 person-years), with 3 (0.6%, 55.2 per 100 000 person-years) of these having died from definite alcohol-related causes (K70.9 alcoholic liver disease, *n* = 1; K74.6 other and unspecified cirrhosis of liver, *n* = 1; K86.0 alcohol-induced chronic pancreatitis, *n* = 1) and 3 (0.6%, 55.2 per 100 000 person-years) from probable alcohol-related causes (K25.4 chronic or unspecified gastric ulcer with hemorrhage, *n* = 1; K26.5 chronic or unspecified duodenal ulcer with perforation, *n* = 1; K76.0 Fatty liver, *n* = 1) (see supplementary table 1). The most common unnatural cause of death was suicide (*n* = 13, 2.4%, 239.1 per 100 000 person-years) (see supplementary table 1). Of the 17 cases with unnatural-cause death for whom we had reliable information on baseline illicit drug use (81.0% of 21 who had died from unnatural causes), 12 (70.6% of 17) had reported at baseline having used illicit drugs in the previous year (cannabis use, *n* = 7 [41.1%]; amphetamine use, *n* = 1 [5.9%]; multiple substance use, *n* = 4 [23.5%]). At follow-up, 3 cases (0.6%, 55.2 per 100 000 person-years) had died from accidental poisoning (heroin intoxication, *n* = 2 [0.4%, 36.8 per 100 000 person-years]; olanzapine and valproate intoxication, *n* = 1 [0.2%, 18.4 per 100 000 person-years]).

Mortality rates by sociodemographic characteristics for all causes, natural causes, and unnatural causes of death are shown in table 1. Mortality rates for all causes, natural causes, and unnatural causes of death were similar in London and Nottingham. All-cause and unnatural-cause mortality rates were lower in women (487.0 per 100 000 person-years) than in men (881.0 per 100 000 person-years), with Kaplan–Meier survival curves and findings from Cox regression suggesting that women experienced a lower risk of unnatural-cause mortality over time than men (see supplementary table 2, supplementary figure 1). Younger cases had lower mortality rates for all causes (466.3 per 100 000 person-years) and natural causes (265.6 per 100 000 person-years). This was also reflected in a shorter time to all- and natural-cause death for older cases (see supplementary table 2, supplementary figure 2). Mortality rates for all causes, natural causes, and unnatural causes of death were slightly lower in cases from a Black and Minority Ethnic (BME) group than in white British cases, but CIs for these point estimates were wide. While there was some limited variation in mortality rates by time since first presentation to services, overall, these were broadly similar over the follow-up period (see supplementary table 3).

**Table 1.** Mortality Rates (per 100 000 person-years) by Baseline Sociodemographic Characteristics

	Cases	All Causes		Natural Causes		Unnatural Causes		Unknown Causes	
		All Deaths	Crude Rate (95% CI)	Natural Deaths	Crude Rate (95% CI)	Unnatural Deaths	Crude Rate (95% CI)	Unknown Deaths	Crude Rate (95% CI)
	*n*	*n* (%)		*n* (%)		*n* (%)		*n* (%)	
Total	549	39 (7.1)	717.3 (524.1–981.8)	15 (2.7)	275.9 (166.3–457.6)	21 (3.8)	386.3 (251.8–592.4)	3 (0.6)	55.2 (17.8–171.1)
Study center									
London	348	23 (6.6)	696.3 (462.7–1047.8)	9 (2.6)	272.5 (141.8–523.7)	13 (3.7)	393.8 (228.5–677.8)	1 (0.3)	30.3 (4.3–214.9)
Nottingham	201	16 (8.0)	749.9 (459.4–1224.0)	6 (3.0)	281.2 (126.3–625.9)	8 (4.0)	374.9 (187.5–749.7)	2 (1.0)	93.7 (23.4–374.8)
Sex									
Women	224	11 (4.9)	487.0 (269.7–879.4)	6 (2.7)	265.6 (119.3–591.3)	4 (1.8)	184.3 (69.2–491.1)	1 (0.5)	44.3 (6.2–314.3)
Men	325	28 (8.6)	881.0 (608.3–1276.0)	9 (2.8)	283.2 (147.3–544.3)	17 (5.2)	534.9 (332.5–860.5)	2 (0.6)	62.9 (15.7–251.6)
Age									
16–29 years	301	14 (4.7)	466.3 (276.2–787.3)	2 (0.7)	66.6 (16.7–266.3)	11 (3.7)	366.4 (202.9–661.6)	1 (0.3)	33.3 (4.7–236.4)
30–65 years	248	25 (10.1)	1027.0 (693.9–1519.9)	13 (5.2)	534.0 (310.1–919.7)	10 (4.0)	410.8 (221.0–763.5)	2 (0.8)	82.2 (20.5–328.5)
Ethnicity									
White British	238	22 (9.2)	919.4 (605.4–1396.2)	8 (3.4)	334.3 (167.2–668.5)	12 (5.0)	501.5 (284.8–883.0)	2 (0.8)	83.6 (20.9–334.2)
BME	311	17 (5.5)	558.5 (347.2–898.4)	7 (2.3)	230.0 (109.6–482.4)	9 (2.9)	295.7 (153.8–568.3)	1 (0.3)	32.9 (4.6–233.2)

*Note*: BME, Black and Minority Ethnic group.

Poisson regression modeling revealed a decreased risk of unnatural-cause mortality in women compared with men after controlling for age at baseline (see table 2). The sex-adjusted rate ratio for the effect of age at baseline indicated significantly reduced risk of all-cause and natural-cause mortality in younger cases. There were no statistically significant differences in risk of mortality by broad ethnic group.

**Table 2.** Rate Ratios (RRs) for All-, Natural-, and Unnatural-Cause Mortality by Baseline Sociodemographic Characteristics

	All Causes				Natural Causes				Unnatural Causes			
	Unadj. RR (95% CI)	*P*	Adj. RR (95% CI)^a^	*P*	Unadj. RR (95% CI)	*P*	Adj. RR (95% CI)^a^	*P*	Unadj. RR (95% CI)	*P*	Adj. RR (95% CI)^a^	*P*
Study center												
London	0.93 (0.49–1.76)	.82	0.89 (0.47–1.68)	.71	0.97 (0.34–2.72)	.95	0.92 (0.33–2.57)	.87	1.05 (0.44–2.53)	.91	1.07 (0.44–2.59)	.87
Nottingham	1.00		1.00		1.00		1.00		1.00		1.00	
Sex												
Women	0.55 (0.28–1.11)	.10	0.50 (0.25–1.01)	.05	0.94 (0.33–2.64)	.90	0.77 (0.27–2.18)	.63	0.33 (0.11–0.98)	.047	0.32 (0.11–0.96)	.04
Men	1.00		1.00		1.00		1.00		1.00		1.00	
Age at baseline												
16–29 years	0.45 (0.24–0.87)	.02	0.42 (0.22–0.82)	.01	0.12 (0.03–0.55)	.01	0.12 (0.03–0.54)	.01	0.89 (0.38–2.10)	.79	0.80 (0.34–1.89)	.61
30–65 years	1.00		1.00		1.00		1.00		1.00		1.00	
Ethnicity												
BME	0.61 (0.32–1.14)	.12	0.62 (0.33–1.17)	.14	0.69 (0.25–1.90)	.47	0.70 (0.25–1.92)	.48	0.59 (0.25–1.40)	.23	0.63 (0.26–1.49)	.29
White British	1.00		1.00		1.00		1.00		1.00		1.00	

*Note*: Abbreviations are explained in the first footnote to table 1.

^a^Adjusted for age at baseline and sex.

### Mortality in the ӔSOP Cohort Compared With the Local General Population

SMRs for all, natural, and unnatural causes of death are shown in table 3. There was an almost 4-fold increase in all-cause mortality in the cohort compared with that in the general population (SMR 3.6, 95% CI 2.6–4.9). All-cause SMRs were of similar magnitude in the 2 study sites (London and Nottingham), slightly more pronounced in men (SMR 4.1, 95% CI 2.8–5.9) than in women (SMR 2.8, 95% CI 1.6–5.1), and lessened for cases in higher age bands. When broken down by natural and unnatural causes of death, the increase in natural-cause mortality was approximately 2-fold, compared with a 13-fold increase in unnatural-cause mortality. Natural-cause SMRs were equally elevated in London and Nottingham as well as in men and women, but not statistically significant at conventional levels (ie, *P* < .05). There was some weak evidence that the increase in natural-cause mortality was higher in age bands 30–44 years and 45–59 years than in age bands 16–29 years and 60–74 years; however, CIs were wide and overlapped across age bands. There was no strong evidence that unnatural-cause SMRs varied by place, sex, or age and these SMRs remained high for older cases (ie, aged 60–74 years). When we further examined risk of suicide as the most common unnatural cause of death in the cohort, this was 20-fold raised compared with that in the local general population (SMR 20.0, 95% CI 11.7–34.5). Although we found no evidence that SMRs for suicide varied by sex and age, there was some evidence that the increase in risk of suicide was more marked in London (SMR 28.3, 95% CI 15.7–51.1) than in Nottingham (SMR 7.7, 95% CI 1.9–30.8), though CIs were very wide and overlapped to a degree.

**Table 3.** Standardized Mortality Ratios (SMRs) for All Causes, Natural Causes, and Unnatural Causes of Death^a^

	All Causes				Natural Causes				Unnatural Causes			
	Observed Deaths	Expected Deaths	SMR	95% CI	Observed Deaths	Expected Deaths	SMR	95% CI	Observed Deaths	Expected Deaths	SMR	95% CI
Total	39	10.8	3.6	2.6–4.9	15	9.1	1.7	1.0–2.7	21	1.6	13.3	8.7–20.4
Study center												
London	23	6.7	3.5	2.3–5.2	9	5.6	1.6	0.8–3.1	13	0.9	14.0	8.2–24.2
Nottingham	16	4.2	3.8	2.3–6.2	6	3.5	1.7	0.8–3.8	8	0.7	12.2	6.1–24.4
Sex												
Women	11	3.9	2.8	1.6–5.1	6	3.6	1.7	0.7–3.7	4	0.3	14.5	5.4–38.7
Men	28	6.9	4.1	2.8–5.9	9	5.5	1.6	0.9–3.2	17	1.3	13.0	8.1–20.9
Age band												
16–29 years	7	1.0	7.4	3.5–15.5	0	0.4	0	—	7	0.5	13.2	6.3–27.8
30–44 years	18	3.1	5.8	3.7–9.2	5	2.3	2.2	0.9–5.3	11	0.8	14.4	8.0–26.1
45–59 years	8	3.3	2.5	1.2–4.9	6	3.0	2.0	0.9–4.5	2	0.2	9.1	2.3–36.6
60–74 years	6	3.5	1.7	0.8–3.8	4	3.5	1.2	0.4–3.1	1	0.1	13.6	1.9–96.8

*Note:* ^a^Indirectly standardized to the age band, sex, and center-specific stratum rates for the population at-risk in Southeast London and Nottinghamshire; SMRs > 1 indicate the magnitude of excess mortality in people with first-episode psychosis.

### All-, Natural- and Unnatural-Cause Mortality by Clinical and Social Factors

There was evidence from Kaplan–Meier survival curves that a long DUP (see supplementary figure 3) and a long time to first remission (see supplementary figure 4) were associated with a shorter time to all- and natural-cause death. Findings from Cox regression further indicated that the association between time to first remission and natural-cause death over time held after controlling for age at baseline and sex (see supplementary table 4). In addition, illicit drug use in the year prior to baseline was associated with a shorter time to all- and unnatural-cause death, while controlling for age and sex (see supplementary table 4, supplementary figure 5). There was also evidence of a longer time to unnatural death for cases with full family involvement at first contact with services (see supplementary table 4, supplementary figure 6).

Rate ratios for all-, natural- and unnatural-cause mortality by clinical and social factors are shown in table 4. While a long DUP was associated with an increased risk of all- and natural-cause mortality in unadjusted analyses, this association was attenuated and ceased to be statistically significant when adjusting for age at baseline and sex. Similarly, after controlling for these variables, the association between a long time to first remission and increased risk of all-cause mortality was reduced and no longer significant. However, the rate ratio for a long time to first remission and increased risk of natural-cause mortality held after controlling for age and sex (see table 4) as well as DUP (LRT, χ^2^ = 0.01, *P* = .92). Further, illicit drug use was associated with a 2- to 3-fold increased risk of all- and unnatural-cause mortality while controlling for age and sex. There was no evidence of confounding of the association between drug use and all-cause mortality by DUP (LRT, χ^2^ = 4.48, *P* = .11), time to first remission (LRT, χ^2^ = 2.31, *P* = .13), and family involvement at first contact (LRT, χ^2^ = 5.27, *P* = .15). However, we found some evidence that the association between illicit drug use and unnatural-cause mortality was confounded by lack of family involvement at first contact (LRT, χ^2^ = 7.22, *P* = .03). Although this association was attenuated, there was still some evidence of an approximately 3-fold increased risk of unnatural-cause mortality in cases using illicit drugs (adj. rate ratio [RR] 3.25, 95% CI 0.96–11.03, *P* = .06). Finally, a significantly reduced risk of unnatural-cause mortality was found in cases with full family involvement compared with those with no family involvement at first contact with services, while controlling for age and sex. This association held when we further adjusted for illicit drug use (χ^2^ = 2.65, *P* = .10).

**Table 4.** Rate Ratios (RRs) for All-, Natural-, and Unnatural-Cause Mortality by Clinical and Social Factors

	All Causes				Natural Causes				Unnatural Causes			
	Unadj. RR (95% CI)	*P*	Adj. RR (95% CI)	*P*	Unadj. RR (95% CI)	*P*	Adj. RR (95% CI)	*P*	Unadj. RR (95% CI)	*P*	Adj. RR (95% CI)	*P*
Clinical factors												
Diagnosis at baseline^a^												
Nonaffective psychosis	0.62 (0.27–1.40)	.25	0.59 (0.26–1.33)	.20	0.42 (0.10–1.87)	.26	0.38 (0.09–1.69)	.20	0.91 (0.33–2.51)	.86	1.04 (0.38–2.89)	.93
Affective psychosis	1.00		1.00		1.00		1.00		1.00		1.00	
DUP^b^												
Long (>2 years)	2.27 (0.99–5.22)	.05	1.66 (0.71–3.87)	.24	3.90 (1.20–12.65)	.02	2.72 (0.83–8.98)	.10	1.75 (0.51–6.06)	.38	1.51 (0.44–5.24)	.51
Short (≤2 years)	1.00		1.00		1.00		1.00		1.00		1.00	
Time to first remission (recovery)^c^												
Long (>2 years)	2.11 (1.02–4.37)	.04	1.91 (0.92–3.99)	.08	6.78 (1.37–33.61)	.02	6.61 (1.33–32.77)	.02	1.13 (0.42–3.01)	.81	1.00 (0.37–2.69)	.99
Short (≤2 years)	1.00		1.00		1.00		1.00		1.00		1.00	
Illicit drug use in year prior to baseline^d^												
Any	1.83 (0.88–3.79)	.11	2.31 (1.06–5.03)	.04	1.02 (0.31–3.33)	.98	2.02 (0.60–6.76)	.25	2.92 (1.03–8.30)	.04	3.78 (1.11–12.89)	.03
None	1.00		1.00		1.00		1.00		1.00		1.00	
Social factors												
Education^e^												
No qualifications	1.37 (0.72–2.63)	.34	1.32 (0.69–2.53)	.41	1.57 (0.57–4.34)	.38	1.46 (0.53–4.03)	.47	1.05 (0.41–2.67)	.92	1.05 (0.41–2.65)	.93
Other	1.00		1.00		1.00		1.00		1.00		1.00	
Employment^f^												
Unemployed	0.70 (0.37–1.33)	.28	0.58 (0.30–1.11)	.10	0.74 (0.31–2.32)	.84	0.75 (0.27–2.07)	.58	0.66 (0.27–1.63)	.37	0.58 (0.23–1.43)	.24
Other	1.00		1.00		1.00		1.00		1.00		1.00	
Involvement of family at first contact^g^												
Full	0.43 (0.19–0.97)	.04	0.49 (0.22–1.11)	.09	0.56 (0.17–1.86)	.35	0.69 (0.21–2.30)	.55	0.09 (0.01–0.66)	.02	0.09 (0.01–0.69)	.02
Limited	0.79 (0.24–2.65)	.70	0.88 (0.26–2.96)	.84	0.69 (0.09–5.54)	.73	0.82 (0.10–6.55)	.85	0.85 (0.19–3.78)	.83	0.89 (0.20–3.94)	.88
None	1.00		1.00		1.00		1.00		1.00		1.00	

*Note*: Adj. RR, adjusted for age at baseline and sex; DUP, duration of untreated psychosis. Missing values: ^a^2, ^b^41, ^c^123, ^d^88, ^e^41, ^f^32, ^g^64.

## Discussion

### Main Findings

This study investigated all-, natural-, and unnatural-cause mortality in a large, epidemiologically characterized cohort of 557 individuals with first-episode psychosis at 10 years follow-up. More cases had died from unnatural than natural causes, with suicide being the leading cause of death. All- and unnatural-cause mortality rates remained broadly similar across the follow-up period. Further, a significantly reduced risk of unnatural-cause mortality was observed in women compared with men. All-cause mortality in the cohort was raised almost 4-fold. When broken down further, an approximately 2-fold increase in natural-cause mortality was observed, compared with a 13-fold increase in unnatural-cause mortality and a 20-fold increase in risk of suicide. The longer the time to first remission, the higher the risk of natural-cause mortality; illicit drug use increased all-cause and (at least partially explained by lack of family involvement at first contact) unnatural-cause mortality risk; and full family involvement at first contact reduced risk of unnatural-cause mortality.

### Methodological Considerations

We investigated the association between baseline clinical and social factors and mortality risk over an approximately 10-year period. However, clinical and social factors may have varied over time. For example, we observed no difference in mortality rates by employment status as assessed at baseline and, even though a large proportion of cases was unemployed at both baseline and follow-up,^28^ this may have varied at the level of individual cases and therefore attenuated differences in mortality rates.^34^ However, restricting our analyses to baseline factors allowed us to establish temporal priority of these overmortality outcome. Further, mortality as an outcome, though markedly elevated compared with the local general population, was, from a purely statistical viewpoint, rare in this cohort, such that CIs were wide, introducing imprecision in point estimates of rate ratios and SMRs. Further follow-up of this cohort would allow us to limit the scope for Type 1 error and ascertain findings from the current set of analyses at 10-year follow-up. In addition, we cannot rule out the possibility of unmeasured confounding by other important factors such as smoking, obesity and other unhealthy lifestyle factors, medication, and access to, or quality of, healthcare.^9,16,19–24^ One of the most important unmeasured factors in the context of this study was alcohol use at baseline. Although this would have allowed us, as for baseline illicit drug use, to investigate its role as a *risk factor* for all-, natural- and unnatural-cause mortality, we were able to examine alcohol use as a *principal underlying cause* of death through the person-tracing procedure by ONS and GRO. Consistent with earlier research^2–6,8–10,12,35–40^ and the classification of principal underlying causes of death by ONS according to ICD-10,^29^ alcohol-related diseases (as the disease which initiated the train of events directly leading to death) were grouped as natural causes, whereas accidental poisoning was classified as unnatural causes of death to enhance comparability of our findings. In interpreting these, it is important to recognize that mortality is likely to be multi-factorial and alcohol and illicit drug use may contribute both as a risk factor several years prior to death, and as a principal underlying cause directly leading to death. However, although our findings are in support of such a role for illicit drug use as both risk factor and principal underlying cause, as well as for alcohol use as a principal underlying cause, no firm conclusions can be drawn about alcohol use as a risk factor for morality. A notable strength of this study is that it was based on a large, epidemiologically characterized cohort of unselected first-episode cases presenting to mental health services within defined catchment areas, for which attrition bias has been reported to be minimal for the 10-year follow-up period.^28^ Given many earlier studies have not been cohorts comprising first-episode cases but included patients with more severe and enduring psychosis, our findings may not be directly comparable with those reported in the wider literature. We were further able to compare mortality in this cohort with that in the local general population of the defined catchment areas, thereby addressing the issue that mortality rates in the general population vary (markedly) by place,^17,18^ which may have obscured earlier reported SMRs quantifying the excess mortality in psychotic disorders.

### Comparison With Previous Research

Over the past decades, consistent evidence has accrued that people with psychotic disorder have higher mortality rates than the general population.^1–9^ Our findings add to this by demonstrating an almost 4-fold increase in all-cause mortality in the ӔSOP cohort compared with the general population. Even though we could not directly compare results between decades, in line with evidence from earlier studies, we noted period effects suggestive of a widening mortality gap, insofar as a greater relative all-cause mortality risk was observed for the decade of follow-up for this cohort than that reported for earlier de- cades.^4,9,12,35,36^ Although the 2-fold increase in natural-cause mortality echoed that of earlier reports, we observed a staggering 13-fold increase in unnatural-cause mortality and a 20-fold increase in risk of suicide, with some evidence of there being a more marked excess of suicide in London than in Nottingham. The SMRs for unnatural-cause mortality (and suicide) that we found in the ӔSOP cohort are markedly higher than the median SMRs of 7.5 for unnatural causes (and 12.9 for suicide) reported in the systematic review by Saha *et al*,^9^ including 3 (10) studies, all conducted in earlier decades.

Although several explanations have been put forward to account for this mortality gap,^9,16,19–24^ only a few studies have investigated the clinical and social factors that may increase risk of premature death in psychosis. We found evidence that a long time to first remission may increase risk of natural-cause mortality in our sample. Although careful replication is required, with account taken of unmeasured confounders and other potentially important explanatory factors, such as suboptimal treatment of physical health problems, engagement with services, and medication use in the time prior to death before firm conclusions can be drawn, it is intriguing to speculate that this finding may point to shared genetic and environmental risk factors indicating severe and enduring psychosis and comorbid somatic conditions.^23,41–43^ Hepgul *et al* ^44^ recently reported increased C-reactive protein levels, as a marker of increased inflammation, and higher body mass index in first-episode psychosis patients exposed to childhood adversity. Given there is also evidence that childhood adversity is associated with an increased risk of developing psychosis,^45^ it may be speculated that elevated inflammation may increase susceptibility to both (enduring) psychotic disorder and metabolic abnormalities, which, in turn, may increase risk of natural-cause mortality. Alternatively, a longer time to remission is likely to involve prolonged periods (and higher dose) of exposure to antipsychotic medication, which have been posited to increase natural-cause mortality via metabolic mechanisms.^46–48^ However, overall, findings on the association between exposure to antipsychotic medication and natural-cause mortality remain inconsistent.^2,48,49^ What is more, when interpreting findings on natural-cause mortality in the ӔSOP cohort, it also needs to be taken into account that at least one-fifth of natural-cause deaths were alcohol-related. In contrast to Neeleman’s^37^ meta-analysis, but echoing more recent studies,^38,39^ we found no difference in all-, natural-, and unnatural-cause mortality between nonaffective and affective psychoses. Consistent with earlier reports,^25,26^ however, at variance with recent findings by Crump *et al*,^2^ our findings suggest that illicit drug use is associated with a 2- to 3-fold increased risk of all- and unnatural-cause mortality. Although 2 cases had died from heroin intoxication and, therefore, some of the elevated risk of unnatural-cause mortality was due to accidental poisoning by this drug, a considerable proportion of cases who had died from unnatural causes at follow-up had reported having used cannabis only at baseline. Notwithstanding that some of the latter may have gone on to use other illicit drugs, this raises the question of alternative explanations for this finding. Indeed, we found some evidence that a lack of family involvement at first contact with services confounded to a degree the association between illicit drug use and unnatural-cause mortality. Although lack of family involvement may have served as a crude proxy of family fragmentation, it remains difficult to disentangle whether it is family fragmentation that may have exacerbated illicit drug use and, in turn, increased risk of unnatural-cause mortality, or vice versa, illicit drug use rendered families more fragmented and, thereby, individuals more vulnerable to death from unnatural causes. It does suggest, however, that these factors may need to be targeted more effectively by mental health services.

It has been repeatedly noted as part of suicide prevention strategies in early psychosis that family members and carers should be closely involved in risk management plans.^50,51^ However, to date, there has been only limited evidence to support this claim. Our finding that family involvement at first contact reduces risk of unnatural-cause mortality, although requiring replication, is, to our knowledge, the first to base family and carer involvement in such prevention strategies on firmer empirical ground. Given, however, that, in line with earlier research,^40^ unnatural-cause mortality rates remained high across the follow-up period, and assuming family involvement may be relevant in reducing unnatural-cause mortality beyond first contact, this may need to be extended to facilitate carer involvement throughout all stages of the illness.^40^

The reduced risk of unnatural-cause mortality that we observed in women compared with men is consistent with most earlier studies investigating this issue to date.^40,52^ Wahlbeck *et al* ^10^ noted that variation in unnatural-cause mortality by sex may result from differences in help-seeking behavior, given women may be more likely than men to talk to health professionals about their mental health. It is also noteworthy that all-cause SMRs lessened with increasing age of cases in our sample, as it suggests that the burden of excess mortality occurs at younger ages and confirms the fact that people with psychosis die younger than the general population. Although mortality risk was slightly lower in cases from the BME compared with the white British group, in contrast to earlier research,^23,24^ we found no difference beyond what would be expected by chance. Consistent with Dickerson *et al*,^23^ the absence of systematic differences across ethnic groups or, as has been reported earlier, an even lower mortality risk in BME groups,^23,24^ may be accounted for by social and health disparities in BME populations being offset through the, relatively speaking, greater attention to medical treatment of physical health problems in patients in the care of mental health services.

## Conclusions

Our findings suggest that the mortality gap in people with schizophrenia and other psychoses remains high and may be wider for unnatural-cause mortality than reported in earlier studies; people with psychosis still do not appear to be benefitting from improvements in healthcare available to the general population.^9,34^ Efforts should now focus on further understanding and targeting these tractable clinical and social risk factors of excess mortality. Early intervention and dual diagnosis services may play a key role in achieving more rapid remission and carer involvement and addressing substance use problems to reduce excess mortality in psychosis. Only then, we expect a narrowing in the mortality gap of psychosis in forthcoming decades.

## Supplementary Material

Supplementary material is available at http://schizophre niabulletin.oxfordjournals.org.

## Funding

This work was supported by UK Medical Research Council (G0500817) and the Department of Health via the National Institute for Health Research (NIHR) Specialist Biomedical Research Centre for Mental Health award to South London and Maudsley NHS Foundation Trust (SLaM) and the Institute of Psychiatry at King’s College London. U.R. is supported by funding from a Postdoctoral Research Fellowship of the UK National Institute of Health Research (NIHR-PDF-201104065) and a Veni grant from the Netherlands Organisation for Scientific Research (451-13-022). R.D. is a Clinician Scientist Fellow of The Health Foundation in partnership with the Academy of Medical Sciences. J.B.K. is supported by a Sir Henry Dale Fellowship jointly funded by the Wellcome Trust and the Royal Society (101272/Z/13/Z). R.M.M. has received honoraria from Janssen, Astra-Zeneca, Lilly, BMS, and Roche. C.M. and R.M.M. are supported by funding from the European Union (European Community’s Seventh Framework Program [HEALTH-F2-2009–241909; Project EU-GEI]). C.M. is further supported by funding from the Wellcome Trust (WT087417). P.B.J. has been a member of scientific advisory boards for Roche and Otsuka during the study.

## Supplementary Material

Supplementary Data
