# Supplementary material for: Mortality in Schizophrenia and Other Psychoses: A 10-Year Follow-up of the ӔSOP First-Episode Cohort
Source: Schizophr Bull. 2014 Sep 27;41(3):664–73. doi: 10.1093/schbul/sbu138 (PMC4393685; doi:10.1093/schbul/sbu138)
Supplement: Supplementary Data [file supp_41_3_664__index.html]

Mortality in Schizophrenia and Other Psychoses: A 10-Year Follow-up of the ӔSOP First-Episode Cohort — Supplementary Data 

# Mortality in Schizophrenia and Other Psychoses: A 10-Year Follow-up of the ӔSOP First-Episode Cohort

## Supplementary Data

Data files

**Files in this Data Supplement:**

- Supplementary Data - Supplementary Data
