# Supplementary material for: Mortality in Schizophrenia and Other Psychoses: A 10-Year Follow-up of the ӔSOP First-Episode Cohort
Source: Schizophr Bull. 2014 Sep 27;41(3):664–73. doi: 10.1093/schbul/sbu138 (PMC4393685; doi:10.1093/schbul/sbu138)
Supplement: Supplementary Data [file supp_sbu138_Mortality_of_schizophrenia_and_other_psychoses___Supplementary_Material___3rd_revision_final.pdf]

Online Supplementary Material

Online Supplementary Tables

Supplementary Table 1. Mortality rates (per 100 000 person-years) by cause of death.

Causes of death (ICD-10 codes)	n (%)	Crude rate	95% CI
**All causes** (A00-R99, U50, V01-Y89)	39 (7.1)	717.3	524.1-981.8
**Natural causes** (A00-Q99)	15 (2.7)	275.9	166.3-457.6
Neoplasms (C00-D48)†	2 (0.4)	36.8	9.2-147.1
Diseases of the circulatory system (I00-I99)‡	4 (0.7)	73.6	27.6-196.0
Diseases of the respiratory system (J00-J99)††	2 (0.4)	36.8	9.2-147.1
Diseases of the digestive system (K00-K95)‡‡	7 (1.3)	128.8	61.4-270.1
**Unknown causes** (R00-R99)	3 (0.6)	55.2	17.8-171.1
**Unnatural causes** (U50.9, V01-Y89)	21 (3.8)	386.3	251.8-592.4
Accidents (V01–X59)	8 (1.5)	147.1	73.6-294.2
Suicide (X60-X84, Y10-Y34)	13 (2.4)	239.1	138.8-411.8

† C18.9 Malignant neoplasm of colon, unspecified (n=1), C92.0 Myeloid leukaemia (n=1)

‡ I25.1 Atherosclerotic heart disease (n=2), I60.9 Nontraumatic subarachnoid haemorrhage, unspecified (n=1), I80.2 Phlebitis and thrombophlebitis of other and unspecified deep vessels of lower extremities (n=1)

†† J18.9 Pneumonia, organism unspecified (n=2)

‡‡ K25.4 Chronic or unspecified gastric ulcer with hemorrhage (n=1), K26.5 Chronic or unspecified duodenal ulcer with perforation (n=1), K63.1 Perforation of intestine (nontraumatic) (n=1), K63.9 Disease of intestine, unspecified (n=1), K70.9 Alcoholic liver disease, unspecified (n=1), K74.6 Other and unspecified cirrhosis of liver (n=1), K76.0 Fatty (change of) liver, not elsewhere classified (n=1), K86.0 Alcohol-induced chronic pancreatitis (n=1)

Supplementary Table 2. Hazard ratios (HRs) for all-, natural- and unnatural-cause mortality over time by baseline socio-demographic characteristics††.

	All causes				Natural causes				Unnatural causes			
	**Unadj. HR (95% CI)**	**p**	**Adj. HR (95% CI) †**	**p**	**Unadj. HR (95% CI)**	**p**	**Adj. HR (95% CI) †**	**P**	**Unadj. HR (95% CI)**	**p**	**Adj. HR (95% CI) †**	**P**
Study centre												
London	0.97 (0.51- 1.85)	0.93	0.93 (0.49-1.76)	0.81	1.06 (0.37-3.02)	0.91	0.98 (0.35-2.78)	0.97	1.03 (0.43-2.49)	0.95	1.05 (0.43-2.55)	0.91
Nottingham	1.00		1.00		1.00		1.00		1.00		1.00	
Sex												
Women	0.55 (0.27-1.11)	0.10	0.50 (0.25-1.01)	0.05	0.92 (0.33-2.59)	0.88	0.77 (0.27-2.18)	0.63	0.33 (0.11-0.99)	0.047	0.32 (0.11-0.97)	0.04
Men	1.00		1.00		1.00		1.00		1.00		1.00	
Age at baseline												
16-29	0.45 (0.23-0.87)	0.02	0.42 (0.22-0.81)	0.01	0.12 (0.03-0.55)	0.01	0.12 (0.03-0.54)	0.01	0.89 (0.38-2.10)	0.79	0.80 (0.34-1.89)	0.61
30-65	1.00		1.00		1.00		1.00		1.00		1.00	
Ethnicity												
BME	0.62 (0.33-1.17)	0.14	0.64 (0.34-1.20)	0.16	0.71 (0.26-1.96)	0.51	0.73 (0.26-2.01)	0.54	0.59 (0.25-1.40)	0.23	0.62 (0.26-1.50)	0.28
White British	1.00		1.00		1.00		1.00		1.00		1.00	

† Adjusted for age at baseline and sex; ††Proportional-hazards assumption based on Schoenfeld residuals

Supplementary Table 3. Mortality rates (per 100 000 person-years) by time since first presentation to services.

	All causes (n=39)			Natural causes (n=15)			Unnatural causes (n=21)		
**Time since first presentation**	**n (%)**	**Crude rate (95% CI)**	**Cum. risk (%) (95% CI)[a]**	**n (%)**	**Crude rate (95% CI)**	**Cum. risk (%) (95% CI)[a]**	**n (%)**	**Crude rate (95% CI)**	**Cum. risk (%) (95% CI)[a]**
0-3 years	14 (2.6)	647.0 (383.2-1092.4)	2.6 (1.5-4.3)	2 (0.4)	92.4 (23.1-369.6)	0.4 (0.0-1.5)	11 (2.0)	508.3 (281.5-917.9)	2.0 (1.1-3.6)
4-6 years	8 (1.5)	519.4 (259.7-1038.5)	1.5 (1.2-1.8)	5 (0.9)	324.6 (135.1-779.8)	1.0 (0.6-1.3)	3 (0.6)	194.8 (62.8-603.9)	0.6 (0.4-0.7)
7-9 years	13 (2.7)	1002.9 (582.3-1727.2)	2.8 (2.2-3.3)	6 (1.3)	482.4 (216.7-1073.7)	1.3 (0.9-1.8)	6 (1.2)	462.9 (207.9-1030.3)	1.3 (1.0-1.7)
10 or more years	4 (1.2)	917.0 (344.2-2443.2)	3.3 (1.9-5.4)	2 (0.6)	458.5 (114.7-1833.2)	1.9 (0.8-4.1)	1 (0.3)	229.2 (32.3-1627.4)	0.6 (0.3-1.2)

[a]Based on Kaplan-Meyer estimates for time period since first presentation

Supplementary Table 4. Hazard ratios (HRs) for all-, natural- and unnatural-cause mortality over time by clinical and social factors††.

	All causes				Natural causes				Unnatural causes			
	**Unadj. HR (95% CI)**	**p**	**Adj. HR (95% CI) †**	**p**	**Unadj. HR (95% CI)**	**p**	**Adj. HR (95% CI) †**	**P**	**Unadj. HR (95% CI)**	**p**	**Adj. HR (95% CI) †**	**P**
**Clinical factors**												
Diagnosis at baseline[a]												
Non-affective psychosis	0.62 (0.27-1.40)	0.25	0.63 (0.28-1.44)	0.27	0.42 (0.09-1.85)	0.25	0.38 (0.09-1.69)	0.20	0.91 (0.33-2.51)	0.86	1.05 (0.38-2.90)	0.93
Affective psychosis	1.00		1.00		1.00		1.00		1.00		1.00	
DUP[b]												
Long (> 2 years)	2.26 (0.99-5.20)	0.05	1.65 (0.71-3.86)	0.25	3.90 (1.20-12.66)	0.02	2.76 (0.84-9.09)	0.10	1.73 (0.50-5.99)	0.38	1.50 (0.43-5.21)	0.52
Short (≤ 2 years)	1.00		1.00		1.00		1.00		1.00		1.00	
Time to first remission (recovery)[c]												
Long (> 2 years)	2.13 (1.03-4.41)	0.04	1.93 (0.93-4.03)	0.08	7.02 (1.42-34.84)	0.02	6.76 (1.36-33.52)	0.02	1.14 (0.43-3.03)	0.80	1.00 (0.37-2.70)	0.99
Short (≤ 2 years)	1.00		1.00		1.00		1.00		1.00		1.00	
Illicit drug use in year prior to baseline[d]												
Any	1.81 (0.87-3.77)	0.11	2.30 (1.06-5.00)	0.04	1.00 (0.31-3.29)	0.99	1.99 (0.59-0.67)	0.27	2.92 (1.03-8.28)	0.04	3.04 (1.00-9.26)	0.05
None	1.00		1.00		1.00		1.00		1.00		1.00	
**Social factors**												
Education[e]												
No qualifications	1.37 (0.72-2.63)	0.34	1.33 (0.69-2.55)	0.39	1.58 (0.57-4.34)	0.38	1.51 (0.55-4.16)	0.43	1.06 (0.42-2.69)	0.90	1.05 (0.41-2.68)	0.91
Other	1.00		1.00		1.00		1.00		1.00		1.00	
Employment[f]												
Unemployed	0.68 (0.36-1.30)	0.25	0.56 (0.29-1.09)	0.09	0.81 (0.29-2.23)	0.68	0.72 (0.26-2.01)	0.54	0.67 (0.27-1.66)	0.39	0.59 (0.24-1.46)	0.25
Other	1.00		1.00		1.00		1.00		1.00		1.00	

† Adjusted for age at baseline and sex; †† Proportional-hazards assumption based on Schoenfeld residuals; Missing values:[a]2, [b]41, [c]123, [d]88, [e]41, [f]32, [g]64

Supplementary Table 4. Hazard ratios (HRs) for all-, natural- and unnatural-cause mortality over time by clinical and social factors†† (continued).

	All causes				Natural causes				Unnatural causes			
	**Unadj. HR (95% CI)**	**p**	**Adj. HR (95% CI) †**	**p**	**Unadj. HR (95% CI)**	**p**	**Adj. HR (95% CI) †**	**P**	**Unadj. HR (95% CI)**	**p**	**Adj. HR (95% CI) †**	**P**
**Social factors**												
Involvement of family at first contact[g]												
Full	0.44 (0.19-0.99)	0.046	0.50 (0.22-1.13)	0.10	0.58 (0.17-1.92)	0.37	0.71 (0.21-2.37)	0.58	0.09 (0.01-0.66)	0.02	0.09 (0.01-0.69)	0.02
Limited	0.80 (0.24-2.68)	0.72	0.89 (0.27-2.99)	0.85	0.70 (0.09-5.58)	0.73	0.83 (0.10-6.65)	0.86	0.86 (0.19-3.80)	0.84	0.90 (0.20-3.99)	0.89
None	1.00		1.00		1.00		1.00		1.00		1.00	

† Adjusted for age at baseline and sex; †† Proportional-hazards assumption based on Schoenfeld residuals; Missing values:[a]2, [b]41, [c]123, [d]88, [e]41, [f]32, [g]64

Online Supplementary Figures

Supplementary Figure 1a. Kaplan-Meier survival curves for risk of death over time (all causes) by sex†.

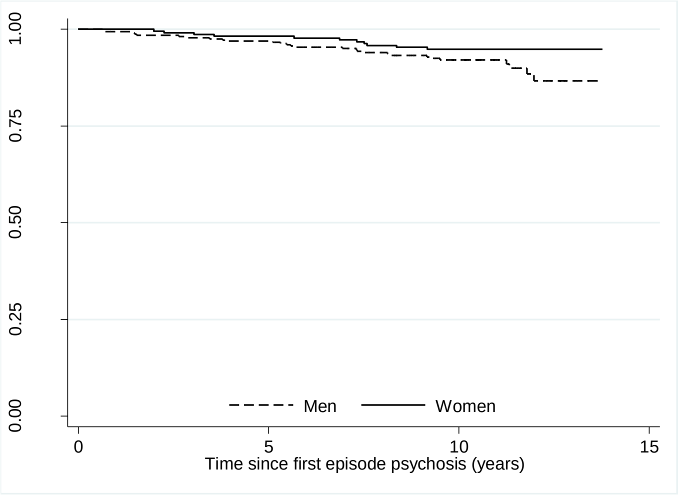

†Log-rank test, χ^2=2.86, p=0.09

Supplementary Figure 1b. Kaplan-Meier survival curves for risk of death over time (unnatural causes) by sex†.

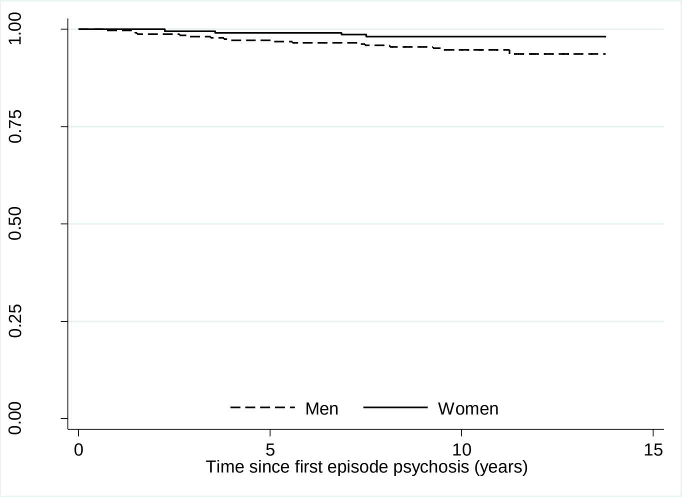

†Log-rank test, χ^2=4.35, p=0.04

Supplementary Figure 2a. Kaplan-Meier survival curves for risk of death over time (all causes) by age†.

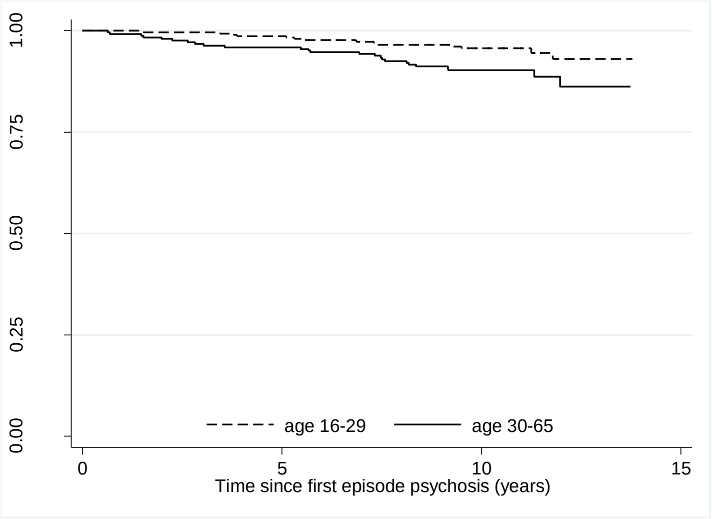

†Log-rank test, χ^2=5.97, p=0.01

Supplementary Figure 2b. Kaplan-Meier survival curves for death risk of death over time (natural causes) by age†.

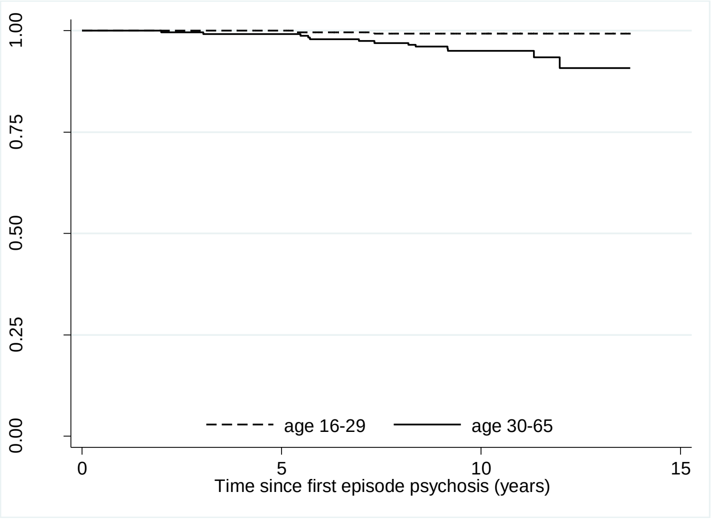

†Log-rank test, χ^2=10.62, p=0.001

Supplementary Figure 3a. Kaplan-Meier survival curves for risk of death over time (all causes) by duration of untreated psychosis (DUP)†.

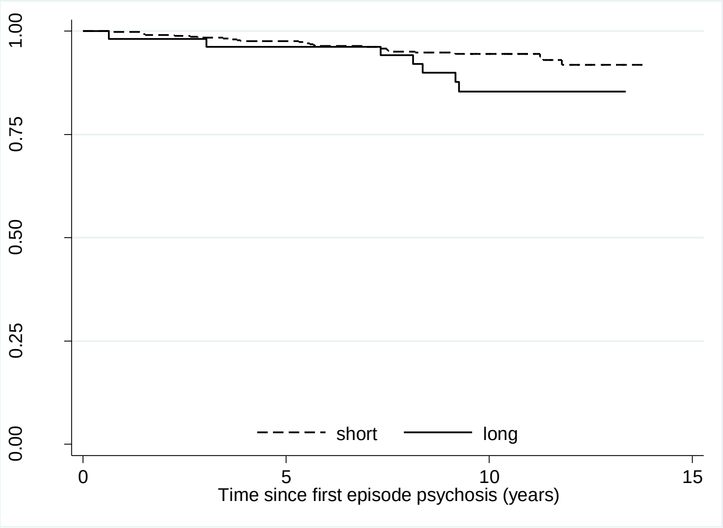

†Log-rank test, χ^2=3.92, p=0.048

Supplementary Figure 3b. Kaplan-Meier survival curves for risk of death over time (natural causes) by duration of untreated psychosis (DUP)†.

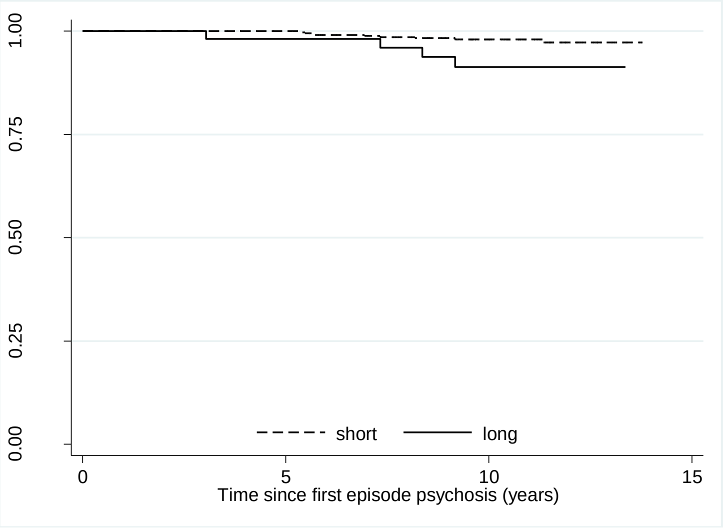

†Log-rank test, χ^2=5.97, p=0.01

Supplementary Figure 4a. Kaplan-Meier survival curves for risk of death over time (all causes) by time to first remission†.

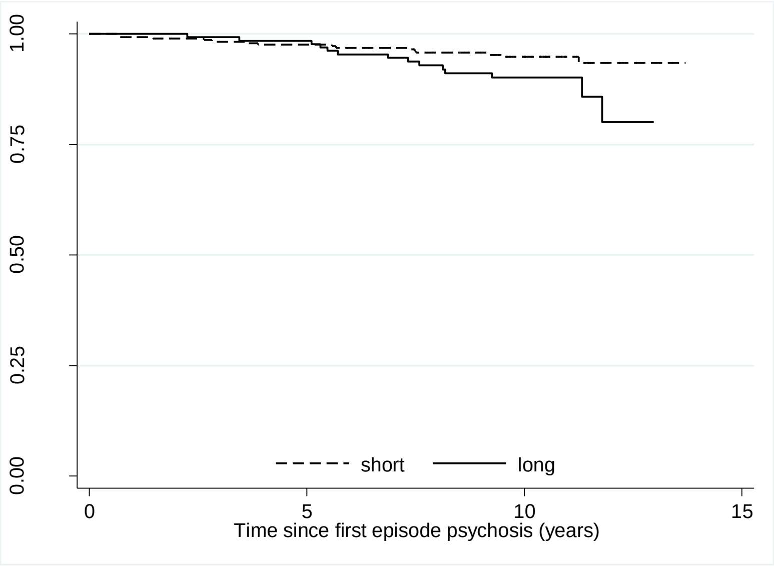

†Log-rank test, χ^2=4.33, p=0.04

Supplementary Figure 4b. Kaplan-Meier survival curves for risk of death (natural causes) over time by time to first remission†.

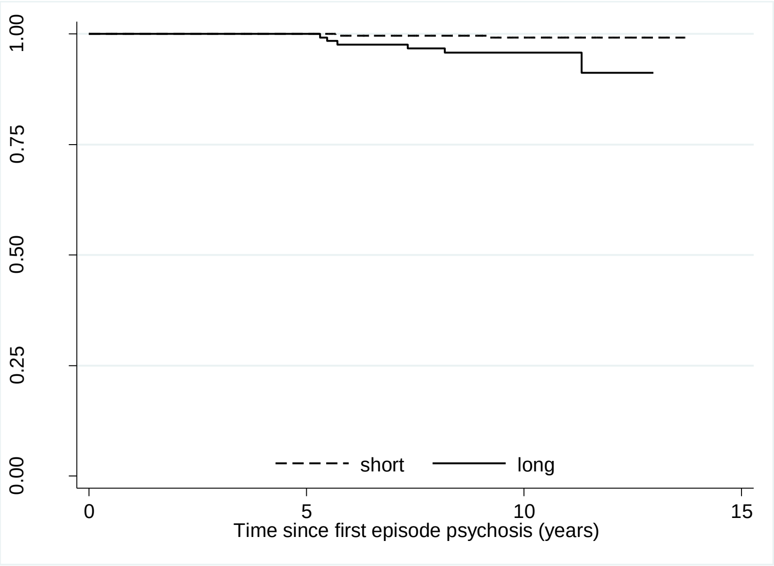

†Log-rank test, χ^2=7.73, p=0.01

Supplementary Figure 5a. Kaplan-Meier survival curves for risk of death over time (all causes) by illicit drug use in year prior to baseline†.

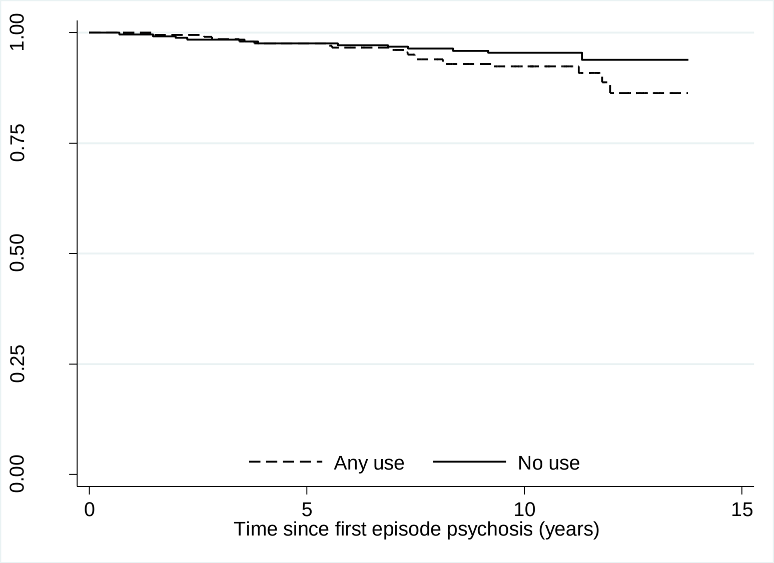

†Log-rank test, χ^2=2.62, p=0.11

Supplementary Figure 5b. Kaplan-Meier survival curves for risk of death over time (unnatural causes) by illicit drug use in year prior to baseline†.

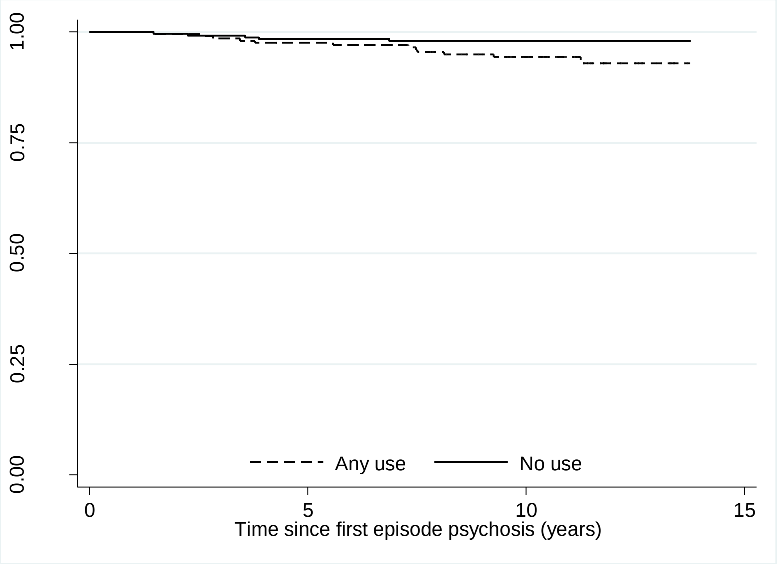

†Log-rank test, χ^2=4.44, p=0.04

Supplementary Figure 6a. Kaplan-Meier survival curves for risk of death over time (all causes) by family involvement at first contact with services†.

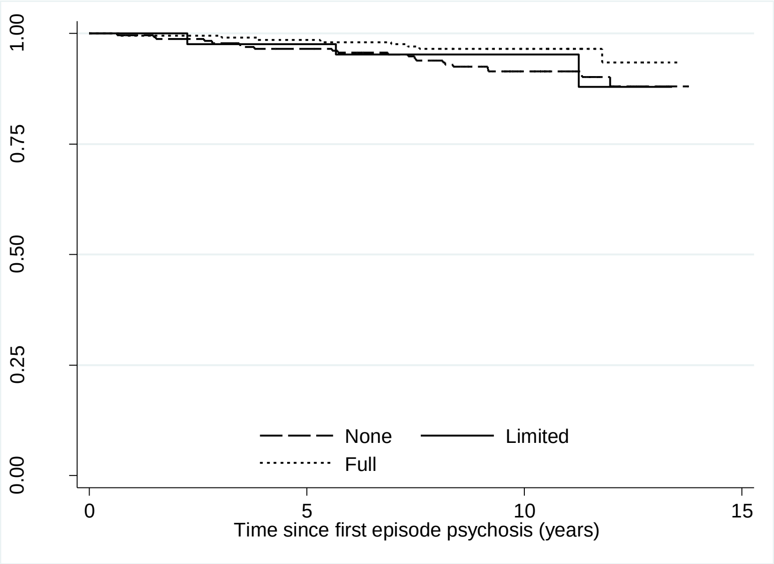

†Log-rank test, χ^2=4.19, p=0.12

Supplementary Figure 6b. Kaplan-Meier survival curves for risk of death over time (unnatural causes) by family involvement at first contact with services†.

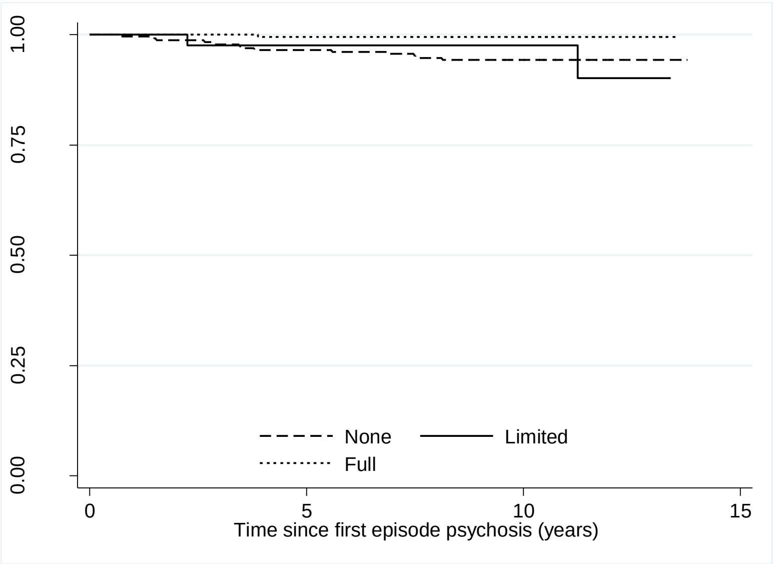

†Log-rank test, χ^2=8.89, p=0.01
